# Distinct Septin Heteropolymers Co-Exist during Multicellular Development in the Filamentous Fungus *Aspergillus nidulans*

**DOI:** 10.1371/journal.pone.0092819

**Published:** 2014-03-24

**Authors:** Yainitza Hernández-Rodríguez, Shunsuke Masuo, Darryl Johnson, Ron Orlando, Amy Smith, Mara Couto-Rodriguez, Michelle Momany

**Affiliations:** 1 Department of Plant Biology, University of Georgia, Athens, Georgia, United States of America; 2 Complex Carbohydrate Research Center, University of Georgia, Athens, Georgia, United States of America; Universidade de Sao Paulo, Brazil

## Abstract

Septins are important components of the cytoskeleton that are highly conserved in eukaryotes and play major roles in cytokinesis, patterning, and many developmental processes. Septins form heteropolymers which assemble into higher-order structures including rings, filaments, and gauzes. In contrast to actin filaments and microtubules, the molecular mechanism by which septins assemble is not well-understood. Here, we report that in the filamentous fungus *Aspergillus nidulans*, four core septins form heteropolymeric complexes. AspE, a fifth septin lacking in unicellular yeasts, interacts with only one of the core septins, and only during multicellular growth. AspE is required for proper localization of three of the core septins, and requires this same subset of core septins for its own unique cortical localization. The Δ*aspE* mutant lacks developmentally-specific septin higher-order structures and shows reduced spore production and slow growth with low temperatures and osmotic stress. Our results show that at least two distinct septin heteropolymer populations co-exist in *A. nidulans*, and that while AspE is not a subunit of either heteropolymer, it is required for assembly of septin higher-order structures found in multicellular development.

## Introduction

Septin GTPases are key components of the cytoskeleton with roles as important and diverse as those of actin, microtubules and intermediate filaments [1]–[3]. Septins form heteropolymers, which then associate into higher-order structures, typically at the cell cortex, where they can act as diffusion barriers, maintaining proteins within discrete subcellular domains, and scaffolds for reorientation of the F-actin cytoskeleton. In contrast with actin filaments and microtubules, very little is known about how septin heteropolymers form or even whether septin function requires these higher-order complexes. Septins play significant roles in developmental processes including polarity establishment, cell division, vesicle trafficking and cell patterning [4], [5]. Perturbation of septins has been associated with fungal pathogenesis in addition to a variety of human diseases such as cancer and Alzheimer's [1]. Septins were first discovered through *S. cerevisiae* mutants that failed to complete cytokinesis [6]. The *S. cerevisiae* septin proteins Cdc3, Cdc10, Cdc11 and Cdc12 were later shown to localize to the yeast neck where they form the 10 nm filament array between the mother cell and daughter bud and serve to restrict and organize cell division proteins at the bud site [4], [5], [7].

The number of septin genes reported in individual organisms ranges from a low of two in *C. elegans* to a high of seventeen in zebrafish [8], [9]. Mammalian septins were placed into four groups based on sequence similarity and named for representative septin members Sept2, Sept3, Sept6 and Sept7 [10]. Subsequent phylogenetic analysis of 78 septins from metazoans confirmed these groups and showed that they were common to vertebrates [9]. Phylogenetic analysis of 162 sequences from animals and fungi placed septins into five groups: Group 1 containing fungal Cdc10 orthologs along with animal Sept3 and Sept6 groups; Group 2 containing fungal Cdc3 orthologs along with animal Sept2 and Sept7 groups; Group 3 containing fungal Cdc11 orthologs; Group4 containing fungal Cdc12 orthologs; and Group 5 containing AspE orthologs exclusively from filamentous fungi [8]. Recently AspE-type septins were also found in the genomes of specific ciliates, diatoms, chlorophyte algae and brown algae, suggesting that this septin-type is likely ancestral and has been lost in multiple lineages [11], [12]. Though AspE-type septins fall into a separate group, they contain the distinct GTP_CDC domain along with other motifs that define septins [8]. While individual phylogenetic analyses differed in the naming of clades and subclades, they consistently group the same septins together.

Studies of Cdc3-, Cdc10-, Cdc11- and Cdc12-type septins (core septins) from fungi and animals have shown that septin monomers associate via two kinds of interfaces (the G and NC interfaces) to form nonpolar heteropolymers. These heteropolymers in turn associate to form higher-order structures that are widely thought to be the biologically active septin form [13]–[15]. Though all of the rules for septin assembly are not yet understood, it is clear that the ability to form dimers via the G or NC interface is important for heteropolymer assembly and that only certain septins can interact with each other. Within a heteropolymer, septins interact either with themselves or with a septin from another group [16], [17].

**Figure 1:**
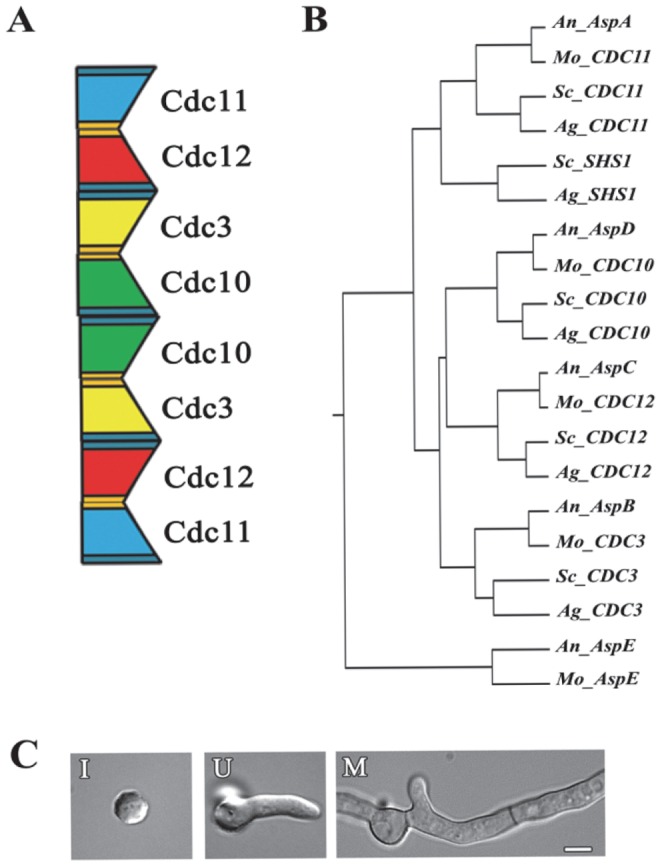
Interactions among *A. nidulans* septins in early development. A) Heterooctamer formation in *S. cerevisiae* vegetative growth (based on Bertin et al, 2008). The wide end of septin subunit (dark blue) represents the NC interface and the narrow end (gold) represents the G interface. B) Clustal W tree of representative fungal septins. Sc_*S. cerevisiae*, Ag_*A. gossypii,* An_*A. nidulans* and Mo_*M. oryzae*. C) *A. nidulans* early developmental stages. I_Isotropic, U_Unicellular polar, and M_Multicellular. Scale bar 5 μm.

In *S. cerevisiae* the major heterooctamer rod in vegetative growth is formed by the core septins in the order Cdc11-Cdc12-Cdc3-Cdc10-Cdc10-Cdc3-Cdc12-Cdc11 (Fig 1A) [13]. The central Cdc10 monomers dimerize via the NC interface and interact with the neighboring Cdc3 monomers via the G interface. Interactions alternate between the NC or G interface along the rest of the heterooctamer rod. Cdc11 in the terminal position of the rod interacts with itself via an NC interface and so connects heterooctamer rods into linear filaments. When the septin Shs1, from the same group as Cdc11, substitutes for Cdc11, heterooctamers associate laterally rather than end-to-end and give rise to a ring rather than a linear filament [18]. If certain septin subunits are eliminated via mutation, new dimer combinations become possible preserving the ability to assemble heteropolymers and higher-order structures [14]. If the central Cdc10 homodimer is eliminated through mutation, the newly exposed Cdc3 subunits homodimerize via the G interface. Similarly, if the terminal Cdc11 subunits are eliminated, the newly exposed Cdc12 subunits homodimerize via the G interface.

The filamentous fungus *Aspergillus nidulans* contains the four core septins, and a fifth septin, the function of which is currently unclear (Fig. 1B). Previous work showed that each of the *A. nidulans* septins could be deleted without loss of viability and led to the hypothesis that the four core septins associate into a heterooctamer in a manner analogous to their *S. cerevisiae* counterparts [19], [20]. The early development of *A. nidulans* from asexual spores (conidia) is naturally synchronous with easily scored developmental landmarks [21]. Here we investigate interactions among the five septins of *A. nidulans* during three distinct stages of early vegetative development: isotropic expansion of the conidium, unicellular polar growth following germ tube emergence, and multicellular growth following septation (Fig. 1C). Using septin fusion proteins expressed from native septin promoters at wild-type levels, immunoprecipitation, and mass spectrometry, we show that the four *A. nidulans* core septins interact as expected during early development, but form distinct stage-specific heteropolymers during multicellular growth. We show that the fifth septin, AspE, localizes to punctate structures at the cell cortex– distinct from higher-order structures typically formed by septins –and, furthermore, is not a member of septin heteropolymers. Mutants lacking *aspE* also show developmental phenotypes during multi-cellular growth under stress conditions. Our results show that at least two distinct septin heteropolymer classes co-exist in *A. nidulans* vegetative growth and that while AspE is not required for the formation of either type of heteropolymer, it is required for the association of one class of heteropolymers into higher-order structures specific to multicellular growth.

## Materials and Methods

### Strains, growth conditions and microscopy

Experiments were carried out using *A. nidulans* strains described in Table S1. Conidia were inoculated to liquid minimal medium (MM) or complete medium (CM) and incubated at 30°C on glass coverslips unless otherwise noted and microscopy was carried out using a Zeiss Axioplan microscope and a Zeiss Axiocam MRc charge-coupled device camera and software as previously described [19], [20]. Photoshop CS3 was used to combine DIC or light images with fluorescence images and for micrograph organization and improvement of contrast and brightness. For plate growth assays, 1×10^3^ freshly harvested conidia of wildtype and Δ*aspE* were inoculated to the center of plates containing MM or CM solid medium with or without 1.2 M sorbitol and incubated at 18°C, 26°C, 30°C, 37°C or 42°C and colony diameter was measured.

### Gene targeting and tagging

Septin gene replacement with *AfpyrG*, *asp-gfp* or *asp-Stag* was by transformation of an *ΔnkuA* strain by cassettes constructed using fusion PCR as previously described [22]. All transformants were verified by diagnostic PCR and Southern hybridization as previously described [19]. Progeny from crosses were verified by diagnostic PCR using KOD XTREME Hot Start DNA Polymerase (71975-3, EMD Millipore) according to manufacturer's instructions.

### Immunoprecipitation and Western blot analysis

1×10^7^ conidia/ml of *A. nidulans* wildtype, septin S-tag, septin-GFP, or septin-GFP, deletion combination strains were incubated in 1–2 L of liquid CM medium with required supplements at 30°C with shaking at 220 rpm for 4–5 h (isotropic), 8–9 h (unicellular) and 16–19 h (multicellular). Cultures at the appropriate developmental stages (verified by microscopy) were harvested, filtered, frozen in liquid nitrogen and ground. Ground cell powder was resuspended in 1 M HK buffer, 300 mM NaCl, 1 mM DTT, with protease inhibitor (Mini, EDTA-free Protease Inhibitor Cocktail Tablets, Roche Applied Science). Cellular debris was removed by centrifugation at 4,000×*g* for 10 mins at 4°C. Supernatant was collected and clarified by two rounds of centrifugation at 14,000×*g* for 10 min at 4°C. Protein in supernatant was quantified with the RC DC Protein Assay Kit (Bio-Rad Laboratories, Hercules, CA) using bovine serum albumin as standard.

For immunoprecipitation, agarose beads conjugated with anti-Stag antibodies (69704, S-protein Agarose, Novagen) or GFP-Trap_A (ChromoTek Gmbh, Planegg-Martinsried, Germany) were combined with *A. nidulans* protein isolated from the appropriate strain, suspended in 1 M HK buffer, 300 mM NaCl, 1 mM DTT with protease inhibitor and incubated for 2 hrs with rocking at 4°C. Samples were washed four times with decreasing volumes of 1 M HK buffer, 300 mM NaCl, 1 mM DTT. Beads were combined with an equal volume of 2× sodium dodecyl sulfate-polyacrylamide gel electrophoresis (SDS-PAGE) sample buffer and samples were treated for 5 min at 95°C.

The solubilized proteins (10–20 μg/lane) were resolved by SDS-PAGE (4% stacking gel, 10% separating gel) on two identical gels. One gel was stained with Coomassie Blue and the other was transferred to a nylon membrane for Western Blotting. The following antibodies were used for Western Blots following manufacturer instructions: Affinity Purified Rabbit anti-Stag, or Affinity Purified Rabbit anti-GFP antibodies 1∶20,000 (Immunology Consultants Laboratory, Portland, OR-USA); Goat anti-Rabbit IgG Horseradish Peroxidase Conjugate 1∶5,000 (CalBiochem/EMD, Billerica, MA-USA). Proteins carrying Stag or GFP fusions were detected using Amersham ECL Western blotting detection reagents and film analysis system according to the manufacturer's instructions (GE Healthcare, United Kingdom).

### LC-MS/MS

Samples were loaded to SDS PAGE gels and run through the stacking gel until samples entered the separating gel, excised, and digested with trypsin. Data was acquired using an Agilent 1100 Capillary LC system (Palo Alto, CA), using the EXP Stem Trap 2.6 μL cartridge packed with Halo Peptide ES-C18 (Optimize Technologies, Oregon City, OR) along with a 0.2×50 mm Halo Peptide ES-C18 capillary column (Advanced Materials Technology, Inc., Wilmington, DE). On-line MS detection used the Thermo-Fisher LTQ ion trap (San Jose, CA) with a Michrom (Michrom Bioresources, Auburn, CA) captive spray interface. Raw tandem mass spectra were converted to mzXML files, then into mascot generic files (MGF) via the Trans-Proteomic Pipeline (Seattle Proteome Center, Seattle, WA). MGF files were searched using Mascot (Matrix Scientific, Boston, MA) against separate target and decoy databases containing only *Aspergillus nidulans* proteins. Normalized spectral counts were produced in ProteoIQ as previously described [23]–[25].

### Clustal Analysis

A rooted phylogenetic tree (UPGMA) was executed using Multiple Sequence Alignment - CLUSTALW: (http://www.genome.jp/tools/clustalw/). Septin sequences from the following organisms were used: Sc_*Saccharomyces cerevisiae*: CDC3/YLR314C, CDC10/YCR002C, CDC11/YJR076C, CDC12/YHR107C, SHS1/YDL225W. Ag_*Ashbya gossypii*: CDC3/AAR111C, CDC10/AAR001CCDC11/AER445C, CDC12/AER238C, SHS1/ABL159W. An_*Aspergillus nidulans*: AspA/AN4667, AspB/AN6688, AspC/AN8182, AspD/AN1394, AspE/AN10595. Mo_*Magnaporthe oryzae*: SEP3/MGG01521, SEP4/MGG06726, SEP5/MGG03087, SEP6/MGG07466 and AspE.

## Results

### The core septins physically interact during early development

Septin monomers bind to each other via their NC and G interfaces to form heteropolymers which interact to form higher-order structures. It is becoming clear that only certain septins are able to bind to each other by a defined NC or G interface and that dimer formation is critical for heteropolymer assembly [16], [17]. The filamentous fungus *A. nidulans* has a single representative of each of the five septin groups, four orthologous to the *S. cerevisiae* core septins and one not found in *S. cerevisiae* (Fig. 1B). To better understand interactions among the five septins of the filamentous fungus *A. nidulans* (AspA-AspE) we performed immunoprecipitation experiments taking advantage of the natural synchrony of easily scored landmarks in early *A. nidulans* development [21]. Unicellular spores (conidia) break dormancy in the presence of a carbon source and have a short period of nonpolar, isotropic expansion (Fig. 1C, I). Isotropically expanding conidia soon establish an axis of polarity and a small protrusion (the germ tube) emerges in unicellular polar growth (Fig. 1C, U). The protruding germ tube continues to extend along the same axis of polarity and is partitioned by crosswalls (septa) forming the multicellular hypha (Fig. 1C, M). Branches emerge from hyphal compartments, with the most basal compartment branching earliest.

**Figure 2:**
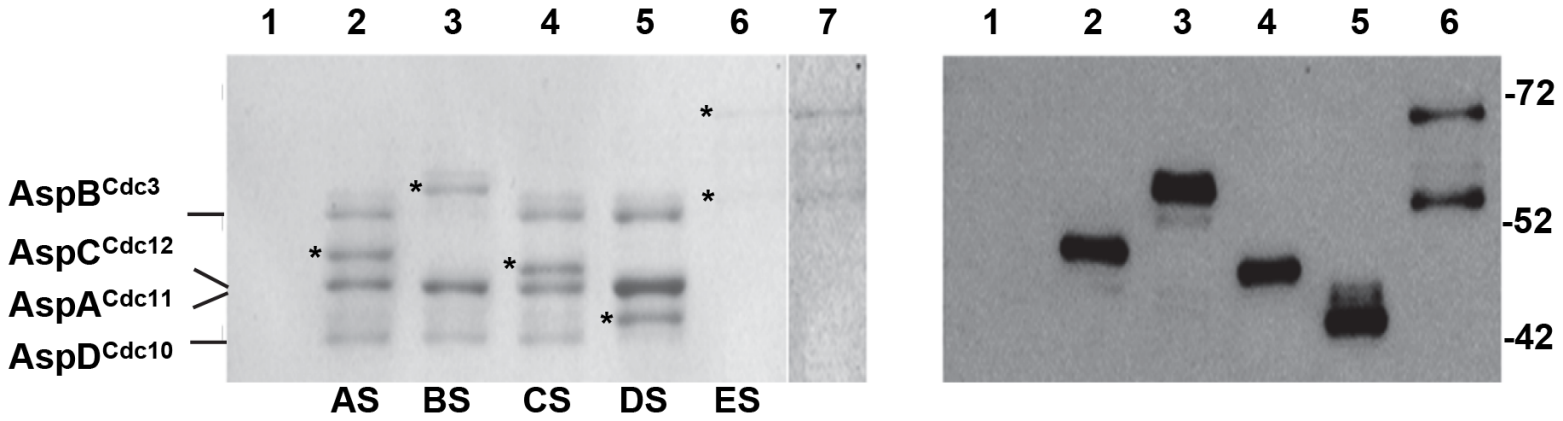
Core septins interact in immunoprecipitation. Protein was isolated from multicellular stage wildtype and strains carrying a single S-tagged septin. Protein was immunoprecipitated by anti-Stag antibodies, separated by SDS PAGE and either stained with Coomassie Brilliant Blue (left panel) or probed with anti S-tag antibodies (right panel). S-tagged septin used as bait in immunoprecipitation is indicated below lane. Asterisk indicates S-tagged septin. Positions of core septins are indicated on the left. Note AspA^Cdc11^ and AspC^Cdc12^ are very close in mass and appear to run as a single protein band. Molecular mass markers are indicated on the right. Lane 1)wildtype; 2) AspA^Cdc11^-Stag strain; 3) AspB^Cdc3^-Stag strain; 4) AspC^Cdc12^-Stag strain; 5) AspD^Cdc10^-Stag strain; 6) AspE^-^Stag strain; 7) Over-exposure of lane 6 to better show immunoprecipitation result. Proteins and predicted mass: AspA^Cdc11^ (43 kDa), AspA^Cdc11^-Stag (AS, 45 kDa), AspB^Cdc3^ (52 kDa), AspB^Cdc3^-Stag (BS, 55 kDa), AspC^Cdc12^ (44 kDa), AspC^Cdc12^-Stag (CS, 46 kDa), AspD^Cdc10^ (39 kDa), AspD^Cdc10^-Stag (DS, 41 kDa), AspE (65 kDa), AspE-Stag (ES, 67 kDa), AspE-Stag smaller band (Es2, ∼55 kDa).

Using fusion PCR and homologous integration [22], we constructed strains in which one of the five septins was replaced with a septin-C-terminal Stag fusion at the corresponding septin locus behind the endogenous promoter. All strains were verified by diagnostic PCR and DNA hybridization and showed wild type morphology. In Western blots, anti-Stag antibodies reacted with bands of the expected mobilities in SDS-PAGE for all five septins but did not recognize proteins in the wild type strain (Fig. S1). To examine interactions among septins, anti-Stag antibodies were used to precipitate proteins from wildtype and Asp-Stag strains at the multicellular stage. Immunoprecipitated proteins were analyzed by SDS-PAGE and Western blot (Fig. 2). As expected, no proteins were precipitated by anti-Stag antibodies from the wildtype strain. In strains carrying either AspA^Cdc11^-Stag, AspB^Cdc3^-Stag, AspC^Cdc12^-Stag or AspD^Cdc10^-Stag, immunoprecipitation pulled down all other untagged core septins, but not AspE. In the strain carrying AspE-Stag, immunoprecipitation from multicellular stage cells resulted in two protein bands differing by approximately 20 kDa. Both bands were recognized by anti-Stag antibodies showing that their C-termini are intact. It is possible that the extra band resulted from proteolytic degradation during protein isolation. It is also possible that the two bands resulted from post-translational modification or alternative splicing, both of which are common among septins [26], [27].

To define the interactions among septins during development, proteins immunoprecipitated from isotropic, unicellular polar and multicellular stages were isolated from SDS-PAGE gels, trypsin digested and analyzed by LC-MS/MS. In strains carrying either AspA^Cdc11^-Stag, AspB^Cdc3^-Stag, AspC^Cdc12^-Stag or AspD^Cdc10^-Stag, immunoprecipitation also pulled down the other three untagged core septins at all developmental stages (Table 1). In contrast, in the strain carrying AspE-Stag, immunoprecipitation pulled down only AspE itself at the isotropic and unicellular polar stages and AspE and low levels of AspB^Cdc3^ at the multicellular stage. No nonseptin proteins were immunoprecipitated from any developmental stage.

**Table 1:** LC-MS/MS Analysis of affinity purified septins.

Dev. Stage	Septin-Stag	Precipitated Septin (normalized spectral counts)				
		AspA	AspB	AspC	AspD	AspE
**Isotropic**	AspA-Stag	40.99	57.71	48.86	32.32	0
	AspB-Stag	23.8	48.7	35.44	37.05	0
	AspC-Stag	22.41	32.72	38.13	27.76	0
	AspD-Stag	28.77	51.34	38.61	48.14	0
	AspE-Stag	0	0.26	0	0	15.08
	WT	0.29	0	0.26	0	0
**Unicellular**	AspA-Stag	43.66	54.49	43.82	36.14	0
**polar**	AspB-Stag	51.34	65.82	49.24	38.65	0
	AspC-Stag	55.08	61.46	56.58	41.19	0
	AspD-Stag	50.53	73.31	53.68	66.43	0
	AspE-Stag	0	1.8	0.26	0.26	28.85
	WT	0	0.61	0.32	0	0
**Multicellular**	AspA-Stag	74.71	148.83	80.93	80.91	0
	AspB-Stag	58.66	156.05	74.27	70.65	0.27
	AspC-Stag	55.29	121.71	66.23	55.00	0
	AspD-Stag	61.81	142.60	79.84	85.31	0
	AspE-Stag	1.615	8.61	1.365	0.26	86.81
	WT	0	1.5	0.32	0.29	2.22

*A. nidulans* wildtype (WT_No Tag) and septin S-tag strains were grown for 4–5 h (isotropic), 8–9 h (unicellular) and 16 h (multicellular) at 30°C in liquid media with shaking. Developmental stages were monitored by microscopy. Proteins were isolated, immunoprecipitated and analyzed by LC-MS/MS. The average of two biological replicates is shown.

### AspE shows punctate localization at the cell cortex and septa during early development

**Figure 3:**
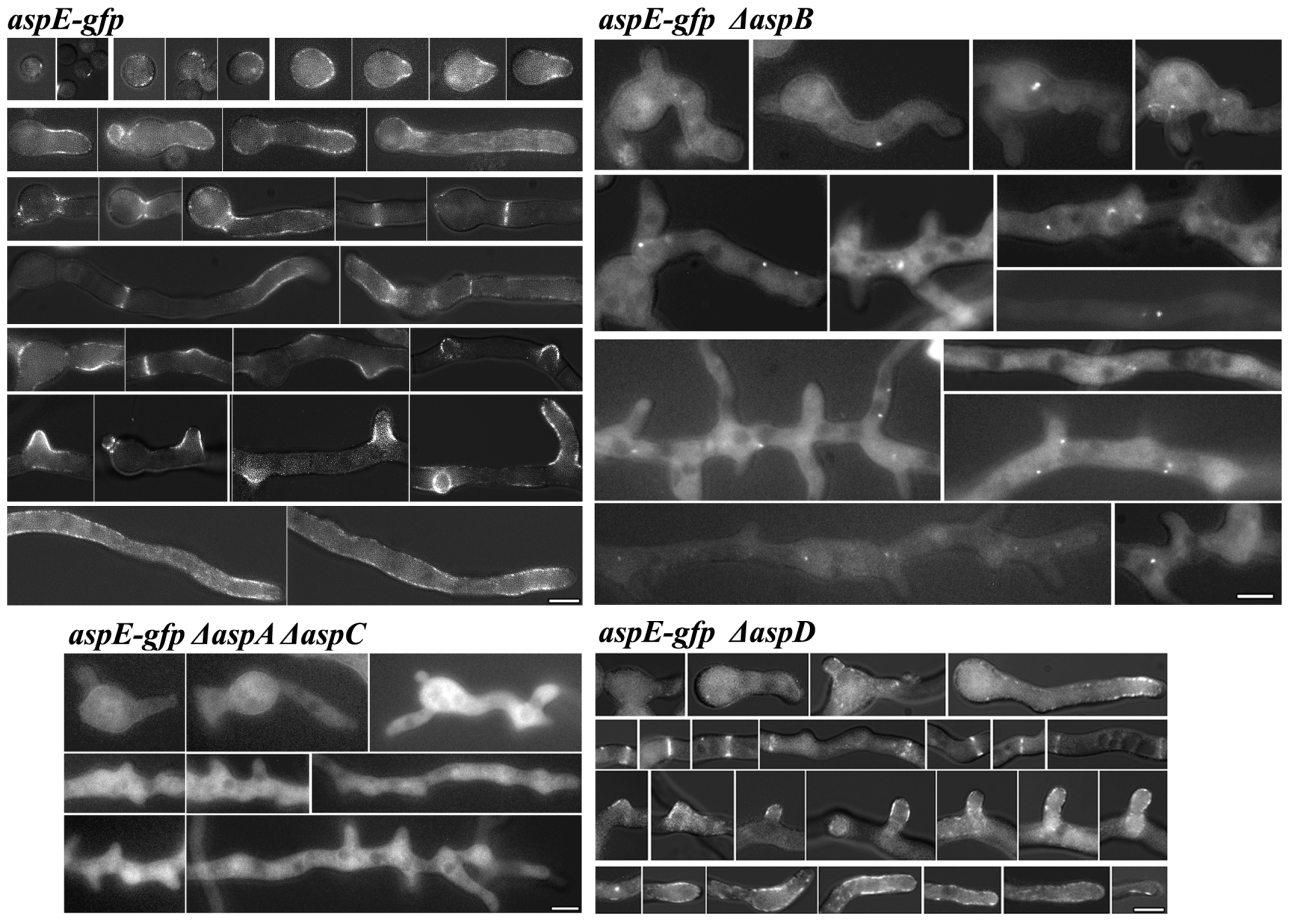
AspE localization is highly cortical and requires AspB^Cdc3^, AspA^Cdc11^ and AspC^Cdc12^, but not AspD^Cdc10^. AspE-GFP is highly cortical throughout early development. Images are arranged chronologically and represent dormant conidia, unicellular polar and multicellular stages of growth. Note *aspE-gfp* Δ*aspA*, *aspE-gfp* Δ*aspC, and aspE-gfp* Δ*aspA*Δ*aspC* strains all showed identical cytoplasmic localization. Scale bar, 5 μm.

In previous work we used homologous integration to construct *A. nidulans* strains in which genes encoding *aspA^CDC11^*, *aspB^CDC3^* or *aspC^CDC12^* were replaced by *asp*-*gfp* fusions [19], [20]. Strains carrying either AspA^Cdc11^-GFP, AspB^Cdc3^-GFP, or AspC^Cdc12^-GFP all showed septin localization as rings at septa and as thick bars and thinner filaments in other areas of the cell during development. To further investigate the roles of AspE, we used homologous integration to construct a strain in which the native *aspE* was replaced by *aspE* fused in frame to *gfp*. *aspE-gfp* was introduced at the *aspE* locus behind the endogenous promoter and the strain was verified by diagnostic PCR and DNA hybridization and showed wild type morphology. AspE-GFP exhibited much weaker fluorescence than the core septins fused to GFP, consistent with earlier results showing that *aspE* RNA is expressed at lower levels than the other septins [28] and the relatively low protein levels seen in immunoprecipitation experiments (Fig. 2). In dormant conidia, AspE localized to the conidial cortex as a prominent single bright spot (Fig. 3). As conidia expanded isotropically, AspE localized as a series of cortical spots and as the germ tube emerged and extended, AspE localization remained cortical. During septum formation, AspE localized to forming septa in a pattern that was more punctate than seen with the other septins. As the hypha continued to extend and branch, AspE remained localized to the cortex of actively growing areas at the hyphal tip and branches. In contrast to the other septins, AspE localization was not detected in conidiophores, the asexual reproductive structures (data not shown).

### AspE localization requires three core septins and localization of these core septins requires AspE

To further investigate the relationship of AspE to the core septins, we constructed strains in which *aspE-gfp* driven by its native promoter at the endogenous locus was combined with each Δ*asp* mutant (Fig. 3). To our surprise, AspE-GFP required all core septins except for AspD^Cdc10^ for normal localization. In the a*spE-gfp* Δ*aspB^cdc3^* strain, AspE localized to the cytoplasm and to a small number of bright spots often at the periphery of nuclei (seen as dark oval areas that exclude AspE-GFP). When combined with Δ*aspA^cdc11^*, Δ*aspC^cdc12^* or Δ*aspA^cdc11^* Δ*aspC^cdc12^*, AspE-GFP localized to the cytoplasm and no punctae or other septin higher-order structures were detected (Fig. 3, and data not shown). In contrast, in the *aspE-gfp* Δ*aspD^cdc10^* strain, AspE localization was punctate at the cell periphery and septa, as seen in the wildtype background (Fig. 3).

**Figure 4:**
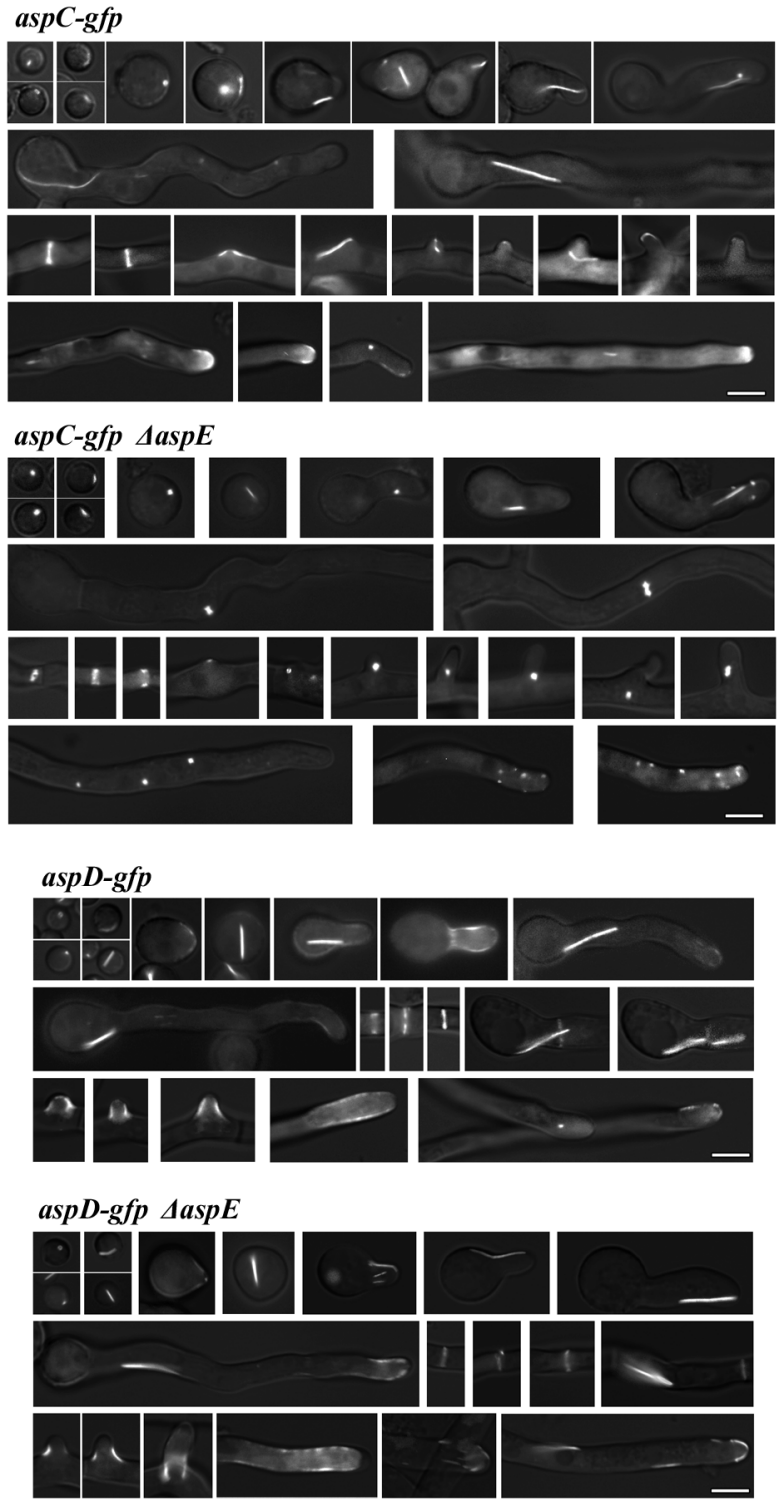
AspE is required for proper AspC^Cdc12^ localization to apical bars, tips of hyphae and branches in the multicellular stage, but not for AspD^Cdc10^ localization. *Top*: AspC^Cdc12^-GFP localizes as dots and bars in dormant conidia and isotropic cells and as bars, filaments, dots, rings, and hyphal caps in unicellular polar and multicellular stages. In Δ*aspE*, AspC^Cdc12^-GFP bars, filaments and caps are lost in the multicellular stage. Scale bar, 5 μm. *Bottom*: AspD^Cdc10^-GFP localizes as dots and bars in dormant conidia and isotropic cells and as bars, filaments, dots, rings, and hyphal caps in unicellular polar and multicellular stages. In Δ*aspE*, AspD^Cdc10^-GFP localization appears unchanged.

In previous work we found that in an Δ*aspE* mutant, AspB^Cdc3^-GFP localization was normal in isotropic and unicellular polar stages, but that in the multicellular stage, it did not form the thick bars or hyphal tip cap seen in wildtype [20]. To determine whether the other core septins require AspE for their localization, we constructed strains in which each *asp-gfp* fusion driven by its native promoter at the endogenous locus was combined with Δ*aspE* (Fig. 4). AspC^Cdc12^-GFP localization in the Δ*aspE* background was normal in isotropic and unicellular polar stages, but in multicellular stage cells the appearance of bars and filaments was greatly reduced and large punctae formed in nascent branches and at hyphal tips. Consistent with previous results showing that AspA^Cdc11^ and AspC^Cdc12^ show identical localization patterns in the wild-type background [19], AspA^Cdc11^ showed the same pattern as AspC^Cdc12^ in the Δ*aspE* background (data not shown). In contrast, AspD^Cdc10^-GFP localized normally in the Δ*aspE* background (Fig. 4).

### AspE is not a member of a septin heteropolymer

**Figure 5:**
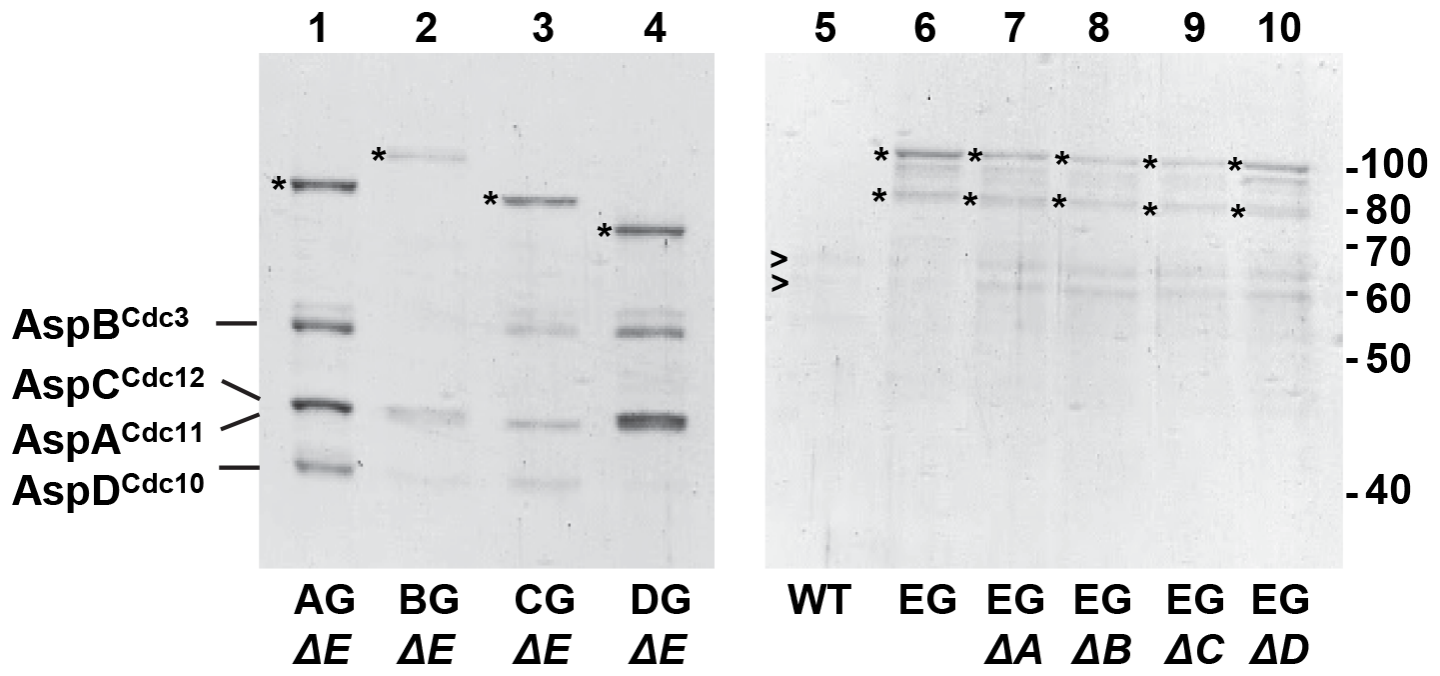
Core septins interact in the absence of AspE. AspE does not substitute for a core septin. Protein was isolated from multicellular stage wildtype and strains carrying a single GFP-tagged septin in combination with a single septin deletion. Protein was immunoprecipitated by anti-GFP antibodies, separated by SDS PAGE and stained with Coomassie Brilliant Blue. GFP-tagged septin used as bait in immunoprecipitation and septin deletion background are indicated below lane. Asterisk indicates GFP-tagged septin. Positions of core septins are indicated on the left and of molecular mass markers on the right. Note, for AspE-GFP immunoprecipitates lanes 5–10, approximately twice the amount of protein was loaded in each lane to improve visualization. Arrowheads indicate nonspecific bands faintly visible in wildtype. Lane 1) *aspA^CDC11^-gfp ΔaspE* strain; 2) *aspB^CDC3^-gfp ΔaspE* strain; 3) *aspC^CDC12^-gfp ΔaspE* strain; 5) *aspD^CDC10^-gfp ΔaspE* strain; 5) wildtype strain; 6) *aspE^-^gfp* strain; 7) *aspE^-^gfp ΔaspA^cdc11^* strain; 8) *aspE^-^gfp ΔaspB^cdc3^* strain; 9) *aspE^-^gfp Δ aspC^cdc12^*strain; 10) *aspE^-^gfp Δ aspD^cdc10^*strain.

We reasoned that the loss of normal AspE-GFP localization in Δ*aspA^cdc11^*, Δ*aspB^cdc3^*, and Δ*aspC^cdc12^* strain backgrounds could have resulted from either failure to assemble heteropolymer units from core septin monomers or failure to assemble heteropolymers into the higher-order structures visualized by fluorescence microscopy. To distinguish between these possibilities, we performed immunoprecipitation experiments on all *aspE-gfp*, Δ*asp* strains in multicellular stage growth (Fig. 5). When any of the GFP-tagged core septins were used as bait in the *ΔaspE* background, all other core septins were precipitated. This suggests that heteropolymers containing the core septins are assembled in the absence of AspE, even though higher-order structures containing AspA^Cdc11^, AspB^Cdc3^, and AspC^Cdc12^ are not visualized without AspE. In reciprocal experiments when AspE-GFP was used as bait in the *Δasp* strains, only AspE was recovered (Fig. 5). This suggests that AspE does not substitute for one of the core septins in an alternate heteropolymer.

### AspE is important for sporulation and multicellular development under stress conditions

**Figure 6:**
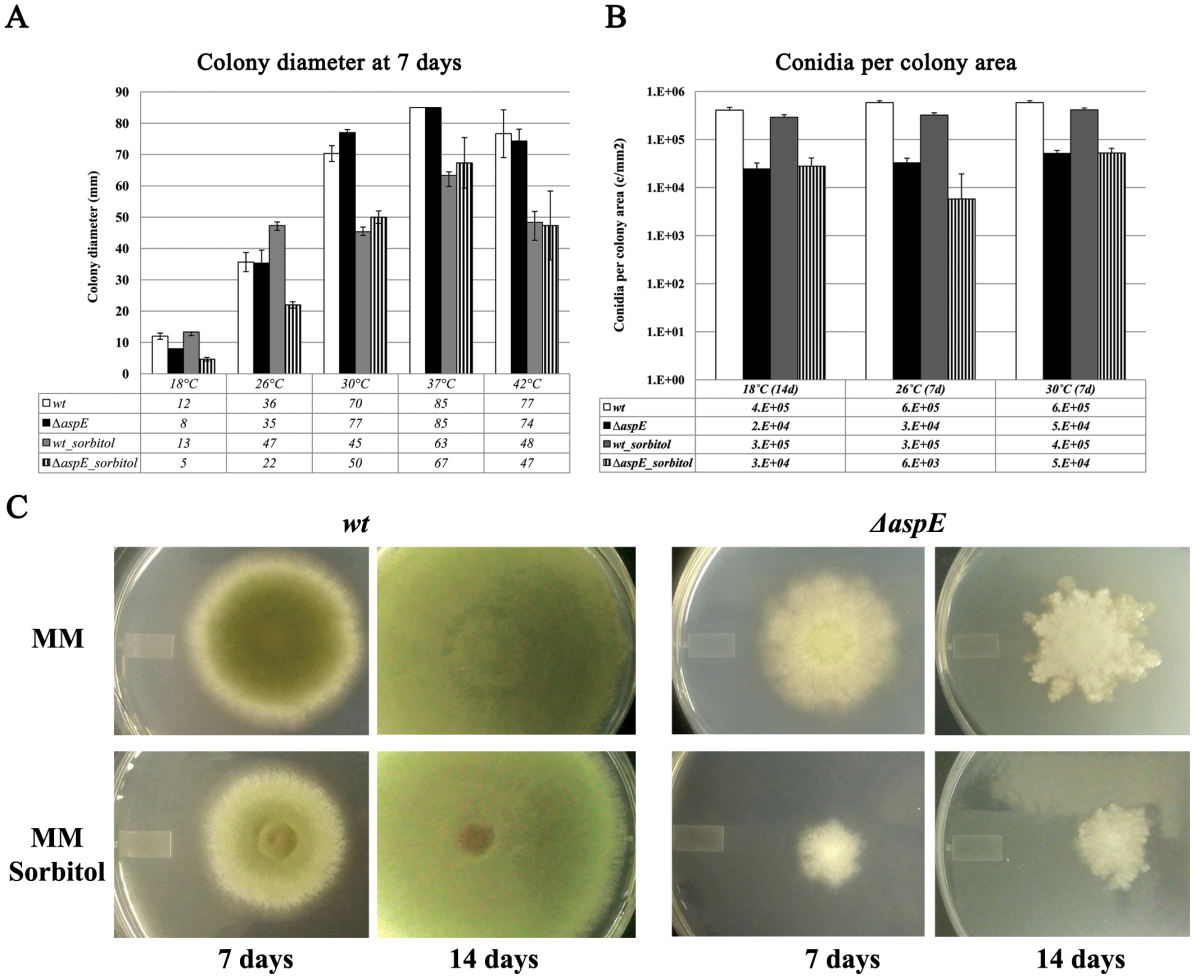
*ΔaspE* shows reduced conidial production and slow growth at low temperatures with increased osmoticum. Wild type and Δ*aspE* strains were inoculated at the same spore concentration to minimal medium (MM) with or without 1.2 M sorbitol and incubated for 7 or 14 days as indicated. A) Plates were incubated for 7 days at 18°C, 26°C, 30°C, 37°C or 42°C, and colony diameter (mm) was measured. Averages were taken from 3 biological replicates. Error bars show standard deviation. B) Plates were incubated for 7 or 14 days. Colony diameter was measured and conidia were harvested in water and counted. Averages were taken from 3 biological replicates. C) Plates were incubated at 26°C for 7 or 14 days as indicated. Representative colony growth is shown. Note irregular colony periphery and shiny appearance typical of colony autolysis seen in Δ*aspE* colonies at 14 days.

To better understand the role of AspE, we examined the growth phenotype of a strain in which *aspE* was replaced by a nutritional marker (Δ*aspE*). The timing and morphology of early developmental landmarks (nuclear division, germ tube emergence, septum formation, and branching) and morphology of conidiophores of the Δ*aspE* strain were virtually indistinguishable from wildtype (Table S2). To examine potential roles of AspE in later growth and under stress, Δ*aspE* and wildtype strains were inoculated to minimal medium with or without 1.2 M sorbitol addition and incubated at temperatures ranging from 18–42°C for 7–14 days (Fig. 6). Conidial production in the *ΔaspE* strain was 8–50-fold lower than in wildtype across all conditions tested. There were few differences in radial expansion of Δ*aspE* and wildtype strains incubated at 30°C or higher. However, when incubation was at 18°C or 26°C with sorbitol in the medium, Δ*aspE* showed much less growth than wildtype. In the most extreme case, when incubated at 18°C on minimal medium supplemented with 1.2 M sorbitol, wildtype grew three times faster than Δ*aspE*. By 14 days *ΔaspE* colonies became shiny with irregular margins, the typical signs of colony autolysis, the enzymatic self-degradation of aging hyphae [29].

## Discussion

**Figure 7:**
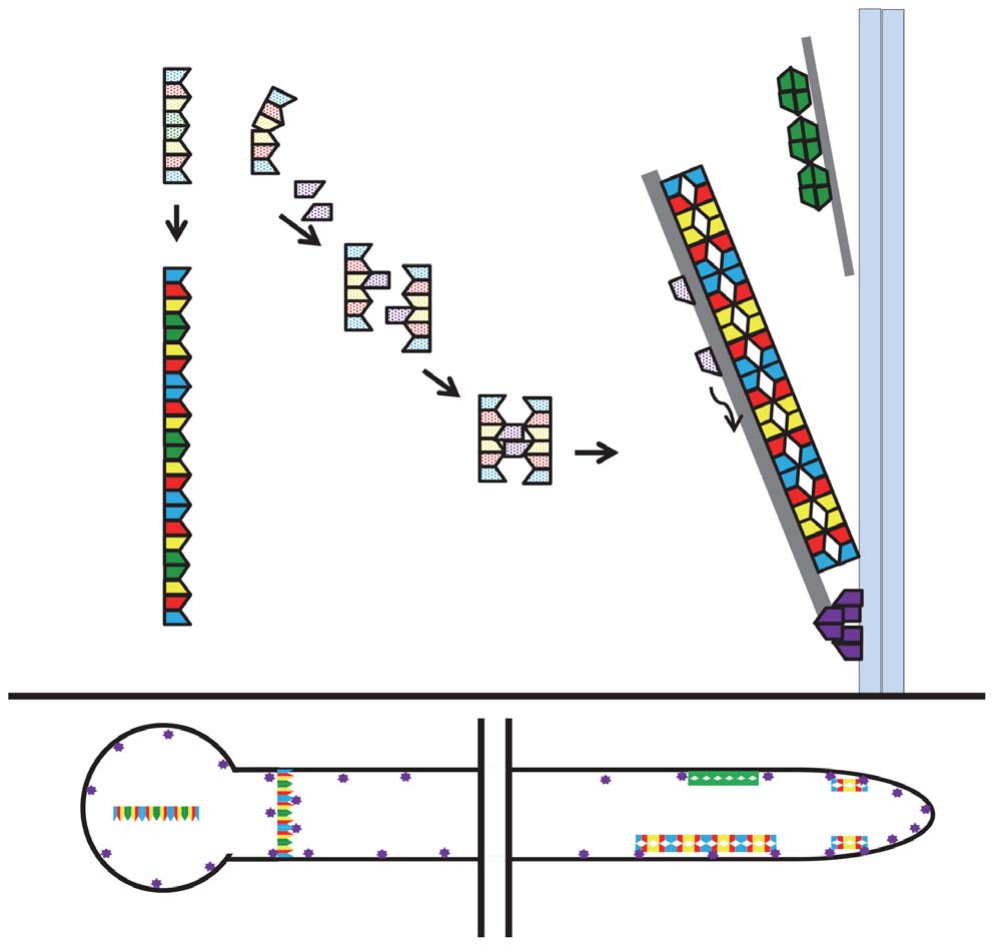
Model of septin heteropolymer and higher-order structure assembly in *A. nidulans*. A) Septin monomers spontaneously form two classes of heteropolymer (indicated by light, stippled colors). Postulated heterooctamers contain all 4 core septins (AspA^Cdc11^, AspB^Cdc3^, AspC^Cdc12^ and AspD^Cdc10^). Postulated heterohexamers lack AspD^Cdc10^, but contain AspA^Cdc11^, AspB^Cdc3^, and AspC^Cdc12^. Classes of heteropolymers have different routes of assembly into the higher-order structures visualized by fluroescence microscopy (indicated by bold, solid colors). Heterooctamers assemble into higher-order structures independently of AspE. Heterohexamers require AspE for assembly into higher-order structures. AspE transiently interacts with heterohexamers via AspB and this transient interaction facilitates higher-order structure assembly. The postulated hexamer higher-order structures stabilize cytoskeletal elements that are required for the delivery of AspE to the plasma membrane where it assembles into cortical higher-order structures. AspD^Cdc10^ also forms higher-order structures at the cortex, possibly by participating in a complex with nonseptins. B) Locations of postulated higher-order structures visualized by fluorescence microscipy within basal (left) and apical (right) regions of the hypha. Heterooctamers form AspE-independent higher-order structures including bars in isotropic and unicellular stages and septal rings in the multicellular stage. Heterohexamers form AspE-dependent higher-order structures including cortical bars and cortical collars and caps at emerging branches and hyphal tips in multicellular stage cells. Stippled, light colors indicate septins within individual heteropolymers. Bold, dark colors indicate septins within higher-order structures that are visualized by fluorescence microscopy. Wavy arrow indicates movement. Light blue double bars indicate plasma membrane. Gray bars indicate cytoskeletal elements. Blue: AspA^Cdc11^; Red: AspC^Cdc12^; Yellow: AspB^Cdc3^; Green: AspD^Cdc10^; Purple: AspE.

Previous studies have demonstrated the presence of septin heterooctamers in fungi and heterohexamers in mammals [13], [14], [16], [17], [30]. Our results show that similar heteropolymer units are formed in *A. nidulans* by core septins AspA^Cdc11^, AspB^Cdc3^, AspC^Cdc12^ and AspD^Cdc10^ throughout early development and that AspE is not a member of these heteropolymers. The apparent nonstoichiometric levels of AspE we observed (Fig. 2) are reminiscent of those reported for the Shs1 septin in *S. cerevisiae* and *A. gossypii* [13], [30]. Bertin et al [13] suggested that Shs1 replaces Cdc11 in a subset of heterooctamers in *S. cerevisiae*. Meseroll et al [30] suggested that septins might form polar heteropolymers with Cdc11 and Shs1 at opposite ends or heterodecamers in *A. gossypii*. However, neither replacement of a core septin in heterooctamers, participation in a polar filament, nor decamer formation appears to explain the relationship of AspE to the other *A. nidulans* septins since none of the S-tagged core septins immunoprecipitated AspE as would be expected if AspE associated with them in a stable heteromeric complex (Fig. 2, Table 1). Similarly, AspE-Stag did not precipitate the core septins except for low levels of AspB^Cdc3^ in the multicellular stage. In *S. cerevisiae*, the association of the Shs1septin into heterooctamers is sensitive to high salt. It is lost from heteropolymers at 1 M KCl, but remains associated at 250 mM [31]. Our immunoprecipitation experiments were done under relatively high ionic strength conditions favoring tightly bound proteins (300 mM NaCl). When we repeated our experiments using AspE-Stag as bait with NaCl concentrations ranging from 100 mM to 300 mM, AspE was still the only protein visible in PAGE (data not shown). While AspE does not appear to be a stable member of a septin heteropolymer, the low levels of AspB^Cdc3^ precipitated by AspE from multicellular stage cells would be consistent with a transient interaction of AspE with septin heteropolymers via AspB^Cdc3^. Interestingly, single AspE group septin genes have been identified in the genomes of the chlorophyte algae *Chlamydomonas reinhardtii, Nannochloris bacillaris* and *Volvox carteri* [11]. Though localization and function studies have not yet been reported for these algal AspE-type septins, since they are singletons, they clearly cannot be members of a canonical septin heteropolymer. Our results also suggest that at least two classes of septin heteropolymers co-exist during *A. nidulans* vegetative development. The first class of heteropolymers contains all four core septins (AspA^Cdc11^, AspB^Cdc3^, AspC^Cdc12^, and AspD^Cdc10^), while the second class contains all core septins except AspD^Cdc10^. Based on known septin heteropolymer organization, we postulate that the first class assembles into heterooctamers while the second class assembles into heterohexamers (Fig. 7, A). Based on immunoprecipitation of septin-GFP fusions in the Δ*aspE* background (Fig. 5), neither postulated heterooctamers nor heterohexamers require AspE for formation. Based on visible fluorescence of Asp-GFP fusions in the Δ*aspE* background, the putative octamer containing all four core septin orthologs (AspA^Cdc11^, AspB^Cdc3^, AspC^Cdc12^, and AspD^Cdc10^) assembles into higher-order structures independently of AspE (Fig. 5, Fig. 7A). We postulate that AspE-independent higher-order structures assembled from putative octamers are seen as spots and bars in isotropic and unicellular stages and as septal rings in the multicellular stage (Fig. 7B). In contrast, the putative hexamer containing AspA^Cdc11^, AspB^Cdc3^, and AspC^Cdc12^ appears to require AspE for assembly into higher-order structures visible by fluorescence microscopy. We postulate that the AspE-dependent higher-order structures assembled from putative hexamers are visible as cortical bars and cortical collars and caps at emerging branches and hyphal tips in multicellular stage cells (Fig.7B). One possible mechanism by which AspE might facilitate higher-order structure assembly of hexamers is via transient association with AspB monomers within the hexamer, leading to a conformational change or stabilization that facilitates assembly into higher-order structures (Fig. 7A). Consistent with a model in which heterohexamers lacking AspD^Cdc10^ participate in different higher-order structures than heterooctamers containing AspD^Cdc10^, the *ΔaspA^cdc11^, ΔaspB^cdc3^*, and *ΔaspC^cdc12^* mutants all show similar perturbations of early developmental landmarks and dramatic emergence of extra germ tubes and branches [19], [20], while the *ΔaspD^cdc10^* mutant shows only subtle thickening and bending of germ tubes and branches (Fig. S2).

The postulated higher-order structures that require AspE for assembly appear to be required for AspE's own localization to visible punctae at the cell cortex. Such a requirement could be explained if the AspE-dependent higher-order structures made from AspA^Cdc11^, AspB^Cdc3^, and AspC^Cdc12^ serve as platforms for assembly of AspE-dependent higher-order structures. Alternatively, perhaps the AspE-dependent higher-order structures stabilize microtubules or microfilaments upon which AspE must assemble or travel to its cortical destination (Fig. 7A). Indeed, interactions of septins with microtubules and microfilaments have been reported in many organisms [2].

In contrast to the core septin orthologs AspA^Cdc11^, AspB^Cdc3^, and AspC^Cdc12^, in the Δ*aspE* background the core septin ortholog AspD^Cdc10^ is still visible in cortical higher-order structures at emerging branches and hyphal tips and as large bars in the cytoplasm (Fig.4), leading us to speculate that AspD^Cdc10^ has another route to the cell cortex (Fig. 7B). Perhaps AspD can stabilize microtubules or microfilaments independently, or perhaps it is part of another complex. More detailed studies of AspD^Cdc10^ are underway to clarify its relationship to the other septins.

AspE orthologs have been localized in two other filamentous fungi with neither reporting the cortical punctae we observed; however in both cases, AspE-GFP was highly overexpressed rather than being driven by the native promoter as in our study. *Aspergillus fumigatus* AspE over-expressed from the *otef* promoter was reported to localize to tubular structures[32] and the *Neurospora crassa* AspE ortholog fused to GFP was localized as an extended hyphal cap at the plasma membrane after overexpression from the *pccg* promoter [33]. Interestingly, when affinity-tagged *N. crassa* septins were expressed from native promoters and used as baits in immunoprecipitation followed by mass spectrometry, core septin orthologs precipitated each other, but not the AspE ortholog, and no nonseptin proteins were recovered, consistent with our results. In contrast when the *A. fumigatus* AspE-GFP overexpressed from the *otef* promoter was used as bait in immunoprecipitation and followed by mass spectrometry, Juvvadi et al [34] reported 48 binding partners, including all other septins, actin, tubulin, heat shock proteins, proteins involved in protein synthesis and over a dozen metabolic proteins.

It is clear that in *A. nidulans* AspE is especially important for the stability of the large septin bars containing AspA^Cdc11^, AspB^Cdc3^, and AspC^Cdc12^ in the multicellular stage. Though the role of these bars is unknown, they seem well-positioned to rigidify the cell cortex or modulate membrane pools, both roles that have been proposed for mammalian septins [1], [35]–[37]. Indeed the hyperbranching phenotype seen in Δ*aspA^cdc11^*, Δ*aspB^cdc3^* and Δ*aspC^cdc12^* [19], [20] is reminiscent of the excessive blebbing and membrane protrusion seen in septin-deficient T cells [38]. Intriguingly, septins have been shown to play an active role in the retraction of membranes during shape change in response to increased hydrostatic pressure in these cells. Perhaps the osmotic sensitivity seen in Δ*aspE* at low temperatures reflects the need for greater cortical stability and membrane modulation with slow growth over long cellular distances. Such a need would be especially pronounced in multicellular organisms and might explain why equivalent septin structures are not seen in the relatively small unicellular fungus *S. cerevisiae*.

## Supporting Information

Figure S1 S-tagged septins from three developmental stages. Total protein isolated from *A. nidulans* wild type and septin S-tagged strains in isotropic, unicellular polar and multicellular stages was separated by SDS PAGE and probed with anti S-tag antibodies.
(TIF)

Figure S2 Δ*aspD^cdc10^* vegetative growth is largely normal and conidiophores are disorganized. *Top*: wildtype early vegetative growth with conidia, unicellular polar and multicellular hyphae. Far right, wildtype conidiophore. *Bottom: Δasp10^cdc10^* shows subtle thickening of germ tube and branch necks, bending of some branches and some swollen tips. Conidiophore layers are highly disorganized. Scale bar, 5 μm.
(TIF)

Table S1 Strains used in this study.
(DOCX)

Table S2 Δ*aspE* is virtually indistinguishable from wildtype in early development. Dormant spores from each strain were stained with Hoechst 33342, and nuclei were counted. After 6 h incubation at 30°C the numbers of germ tubes were counted. For septum positioning, distance from the closest septum to the conidial compartment was measured. For branch number, after incubation for 12h at 30°C, branches per compartment delineated by two septa were counted. For conidiophore morphology, spores were incubated in agar between coverslips for 3 days at 30°C. Conidiophores were categorized as normal if all layers were present and abnormal if layers were absent or aberrant. The average of two independent replicates is shown. STDV, standard deviation.
(DOCX)
