# Supplementary figures and images for: Distinct Septin Heteropolymers Co-Exist during Multicellular Development in the Filamentous Fungus Aspergillus nidulans
Source: PLoS One. 2014 Mar 24;9(3):e92819. doi: 10.1371/journal.pone.0092819 (PMC3963935; doi:10.1371/journal.pone.0092819)

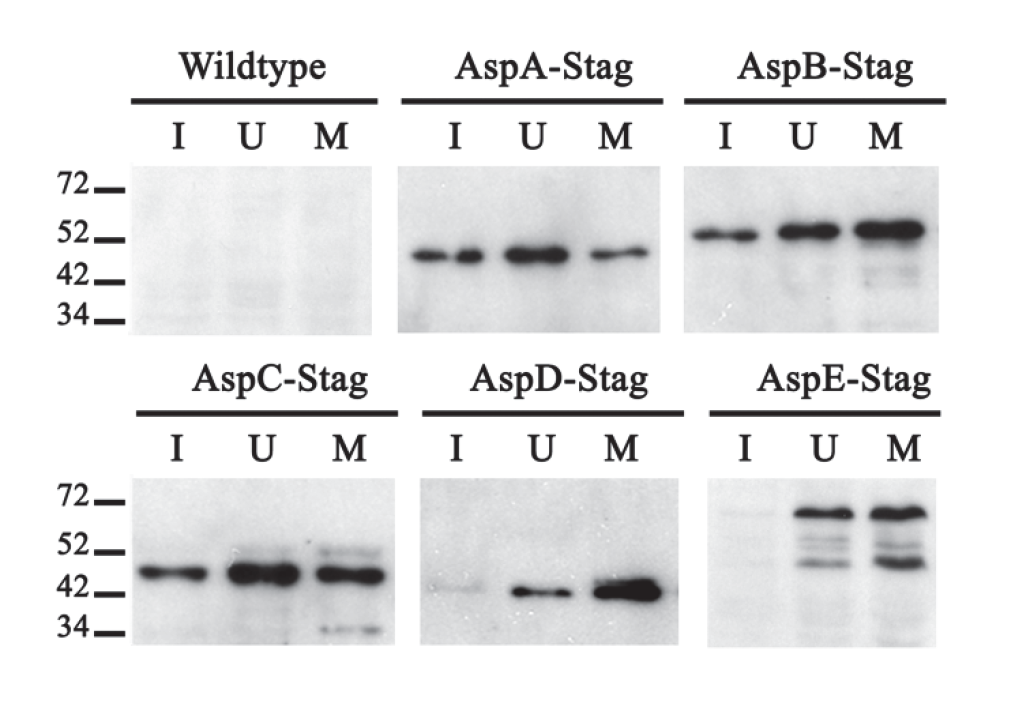

Supplement: Figure S1 — S-tagged septins from three developmental stages. Total protein isolated from A. nidulans wild type and septin S-tagged strains in isotropic, unicellular polar and multicellular stages was separated by SDS PAGE and probed with anti S-tag antibodies. (TIF) [file pone.0092819.s001.tif]

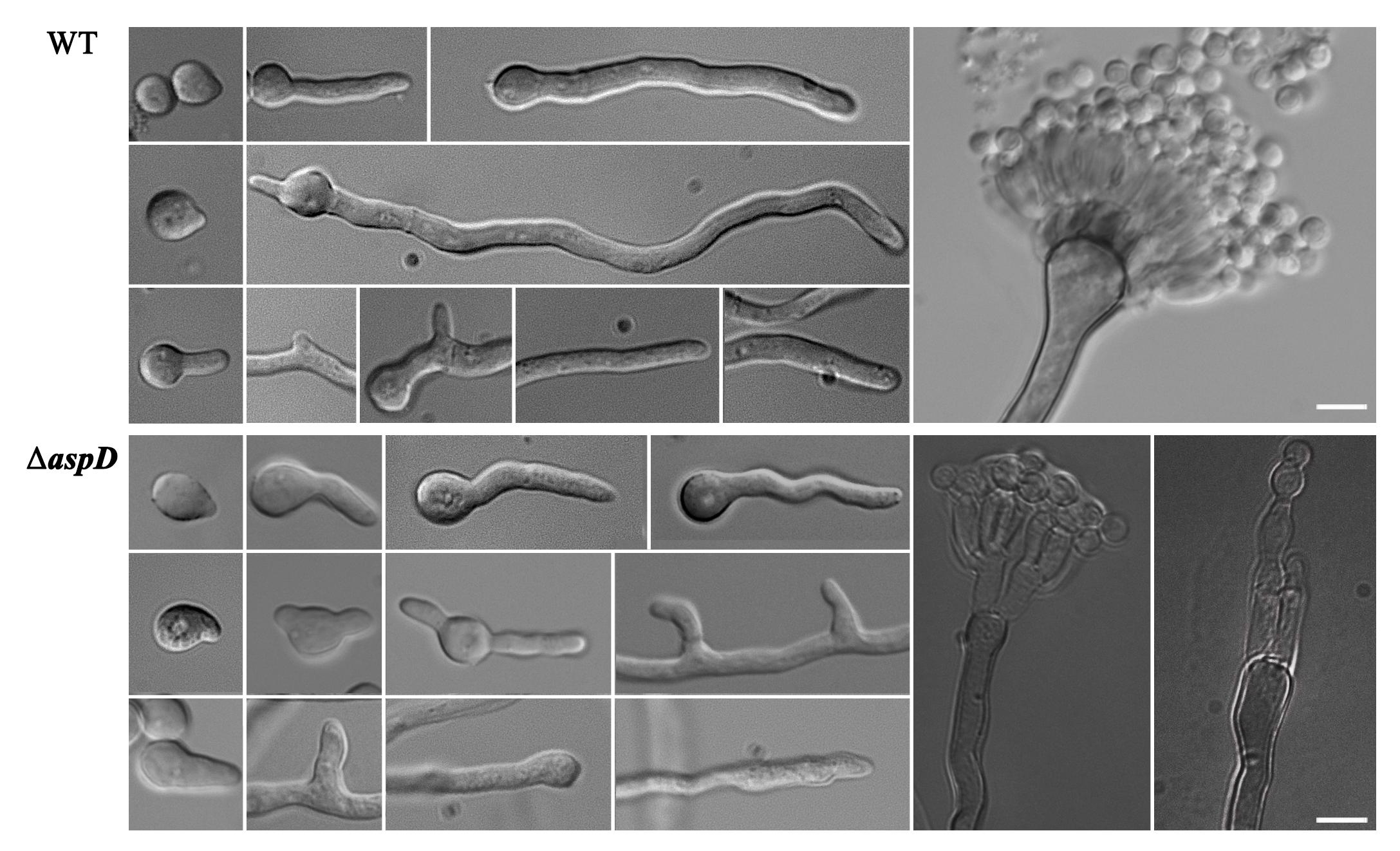

Supplement: Figure S2 — ΔaspDcdc10 vegetative growth is largely normal and conidiophores are disorganized. Top: wildtype early vegetative growth with conidia, unicellular polar and multicellular hyphae. Far right, wildtype conidiophore. Bottom: Δasp10cdc10 shows subtle thickening of germ tube and branch necks, bending of some branches and some swollen tips. Conidiophore layers are highly disorganized. Scale bar, 5 μm. (TIF) [file pone.0092819.s002.tif]
