# Supplementary material for: Distinct Septin Heteropolymers Co-Exist during Multicellular Development in the Filamentous Fungus Aspergillus nidulans
Source: PLoS One. 2014 Mar 24;9(3):e92819. doi: 10.1371/journal.pone.0092819 (PMC3963935; doi:10.1371/journal.pone.0092819)
Supplement: Table S1 — Strains used in this study. (DOCX) [file pone.0092819.s003.docx]

**Table S1. Strains used in this study**

| Strain | Genotype | Source or reference |  |
| --- | --- | --- | --- |
| A850 | *biA1; argB::trpC_B; methG1; veA1; trpC801* | FGSC |  |
| A1145 | *pyrG89; pyroA4; nkuA::argB; riboB2* | FGSC |  |
| A1147 | *pyrG89; argB2; pabaB22; nkuA::argB; riboB2* | FGSC |  |
| AAS002 | *aspA::aspA-gfp-AfpyrG; aspE::AfpyrG* | This study |  |
| AKK3 | *aspD::AfpyrG; pyroA4; argB2* | This study |  |
| ARL96 | *pyrG89; pyroA4; nkuA::argB; riboB2; aspA::aspA-stag-AfpyrG* | This study |  |
| ARL108 | *pyrG89; pyroA4; nkuA::argB; riboB2; aspC::aspC-stag-AfpyrG* | This study |  |
| ARL141 | *aspA::aspA-gfp-AfpyrG; argB2; methG1* | Lindsey et al 2009 |  |
| ARL159 | *aspC::AspC-gfp-AfpyrG; pabaA6* | Lindsey et al 2009 |  |
| ASH6 | *pyrG89; pyroA4; riboB2; aspE::aspE-stag-AfpyrG* | This study |  |
| ASH27 | *aspD::aspD-gfp-AfpyrG; argB2; riboB2* | Hernandez-Rodriguez et al 2012 |  |
| ASH41 | *aspE::AfpyrG; riboB2* | Hernandez-Rodriguez et al 2012 |  |
| ASH42 | *aspE::aspE-gfp-AfpyrG; riboB2* | This study |  |
| ASH43 | *aspE::AfpyrG; pyroA4* | This study |  |
| ASH45 | *aspE::aspE-gfp-AfpyrG* | This study |  |
| ASH85 | *aspE::aspE-gfp-AfpyrG; aspB::AfpyrG* | This study |  |
| ASM1 | *pyrG89; pyroA4; wA3; aspD::aspD-stag-AfpyrG* | This study |  |
| AYR4 | *pyrG89; pyroA4; riboB2; aspB::aspB-stag-AfpyrG* | This study |  |
| AYR6 | *aspB::aspB-gfp-AfpyrG; pyrG89; argB2* | Hernandez-Rodriguez et al 2012 |  |
| AYR26 | *aspB::aspB-gfp-AfpyrG; aspE::AfpyrG* | Hernandez-Rodriguez et al 2012 |  |
| AYR30 | *aspB::AfpyrG; pyroA4; argB2* | This study |  |
| AYR82 | *aspE::aspE-gfp-AfpyrG; aspA::argB2; aspC::AfpyrG* | This study |  |
| AYR83 | *aspE::aspE-gfp-AfpyrG; aspC::AfpyrG* | This study |  |
| AYR84 | *aspE::aspE-gfp-AfpyrG; aspA::argB2* | This study |  |
| AYR85 | *aspE::aspE-gfp-AfpyrG; aspD::AfpyrG* | This study |  |
| AYR91 | *aspC::aspC-gfp-AfpyrG; aspE::AfpyrG* | This study |  |
| AYR92 | aspD::aspD-gfp-AfpyrG; aspE::AfpyrG | This study |  |
