# Supplementary material for: Distinct Septin Heteropolymers Co-Exist during Multicellular Development in the Filamentous Fungus Aspergillus nidulans
Source: PLoS One. 2014 Mar 24;9(3):e92819. doi: 10.1371/journal.pone.0092819 (PMC3963935; doi:10.1371/journal.pone.0092819)
Supplement: Table S2 — ΔaspE is virtually indistinguishable from wildtype in early development. Dormant spores from each strain were stained with Hoechst 33342, and nuclei were counted. After 6 h incubation at 30°C the numbers of germ tubes were counted. For septum positioning, distance from the closest septum to the conidial compartment was measured. For branch number, after incubation for 12h at 30°C, branches per compartment delineated by two septa were counted. For conidiophore morphology, spores were incubated in agar between coverslips for 3 days at 30°C. Conidiophores were categorized as normal if all layers were present and abnormal if layers were absent or aberrant. The average of two independent replicates is shown. STDV, standard deviation. (DOCX) [file pone.0092819.s004.docx]

**Table S2. Δ*aspE* is virtually indistinguishable from wildtype in early development.**

| Average nuclear number [n=100 (2X)] | | | | | | | | | | | | | | | | | | | | | | | | |
| --- | --- | --- | --- | --- | --- | --- | --- | --- | --- | --- | --- | --- | --- | --- | --- | --- | --- | --- | --- | --- | --- | --- | --- | --- |
| Strain | 0 nucleus | | STDV | | 1 nucleus | | | STDV | | | 2 nuclei | | | STDV | | | | | 4 nuclei | | | | STDV | |
| wt | 5% | | 0 | | 87% | | | 0 | | | 8% | | | 0 | | | | | 0% | | | | 0 | |
| *ΔaspE* | 7% | | 0 | | 86% | | | 0 | | | 8% | | | 0 | | | | | 0% | | | | 0 | |
| Average germ tube (gt) number [n=100 (2X)] | | | | | | | | | | | | | | | | | | | | | | | | |
| Strain | 0 gt | | STDV | | 1 gt | | | STDV | | | 2 gt | | | | STDV | | | | | 3 gt | | | STDV | |
| wt | 94% | | 0 | | 3% | | | 0 | | | 0% | | | | 0 | | | | | 0% | | | 0 | |
| *ΔaspE* | 80% | | 0.1 | | 18% | | | 0.1 | | | 1% | | | | 0 | | | | | 0% | | | 0 | |
| Average of first septum position in reference to conidial compartment [n=100 (2X)] | | | | | | | | | | | | | | | | | | | | | | | | |
| Strain | No septum | | | | STDV | | At 0-5µm | | | | | | STDV | | | | At >5µm | | | | | STDV | | |
| wt | 10% | | | | 0 | | 86% | | | | | | 0 | | | | 4% | | | | | 0 | | |
| *ΔaspE* | 10% | | | | 0.1 | | 88% | | | | | | 0 | | | | 2% | | | | | 0 | | |
| Average branch (br) number [n=100 (2X)] | | | | | | | | | | | | | | | | | | | | | | | | |
| Strain | 0 br | STDV | | 1 br | | STDV | | | 2 br | | | STDV | | | | 3br | | STDV | | | ≥4br | | | STDV |
| wt | 75% | 0.1 | | 24% | | 0 | | | 1% | | | 0 | | | | 0% | | 0 | | | 0% | | | 0 |
| *ΔaspE* | 54% | 0.2 | | 43% | | 0.2 | | | 3% | | | 0 | | | | 0% | | 0 | | | 0% | | | 0 |
| Average Conidiophore morphology [n=100 (2X)] | | | | | | | | | | | | | | | | | | | | | | | | |
| Strain | Normal | | | STDV | | | | | | Abnormal | | | | | | | | STDV | | | | | | |
| wt | 95% | | | 0 | | | | | | 6% | | | | | | | | 0 | | | | | | |
| *ΔaspE* | 88% | | | 0.1 | | | | | | 12% | | | | | | | | 0.1 | | | | | | |
